# A Chinese female patient with LGI1 and mGluR5 antibodies: A case report

**DOI:** 10.1097/MD.0000000000031063

**Published:** 2022-10-28

**Authors:** Tiantian Huo, Xintong Luo, Jingru Zhao, Tianjun Wang, Jinghong Chen

**Affiliations:** a Department of Neurology, Hebei General Hospital, Shijiazhuang, China.

**Keywords:** anti-LGI1 encephalitis, anti-mGluR5 encephalitis, case report

## Abstract

Anti-LGI1 antibody encephalitis and anti-mGluR5 are both uncommon encephalitis, and we report the first case of autoimmune encephalitis (AE) with dual seropositive antibodies of leucine-rich glioma-inactivated 1 (LGI1) and mGluR5.

**Patient concerns:**

We present a case of AE with dual seropositive antibodies of LGI1 and mGluR5 in a 65-year-old woman who presented with sudden onset left faciobrachial dystonic seizures and unresponsive for 5 hours.

**Diagnosis:**

The patient was diagnosed with anti-LGI1 AE and anti-mGluR5 AE mainly based on the clinical symptoms and further test of the antibody in serum and cerebral spinal fluid (CSF).

**Interventions and outcomes:**

The patient was treated with glucocorticoid intravenous drip. We also gave her the therapy of immunoglobulin (25 g q.d) for 5 days and anti-epileptic therapy. She had no more convulsions on the left side of the face and limbs. She did not complain of any uncomfort until July 18.

**Lessons:**

Early recognition of AE is crucial. Specific autoantibodies are associated with corresponding syndromes. Our patient was initially diagnosed with acute ischemic stroke. Therefore, we should conduct further study on the related symptoms of AE.

## 1. Introduction

Anti-leucine-rich glioma inactivated protein 1 (LGI1) encephalitis is the second most common autoimmune encephalitis (AE). It is a common type of the voltage-gated potassium channel complex (VGKC-complex) antibody encephalitis^[1]^ which is diagnosed mainly by serum/CSF LGI1 antibodies, five core clinical syndromes (facial-brachial dystonia episodes, refractory hyponatremia, cognitive impairment, epilepsy, psychiatric symptoms). The brain MRI may show bilateral or unilateral abnormal signal in the medial temporal lobe or basal ganglia, especially in T2-weighted sequence of hyperintensity, or no obvious abnormality.^[2]^ Peripheral nerve hyperexcitability syndrome (PNHS) may observed in anti-LGI1 AE patients.^[3]^

Anti-metabotropic glutamate receptor 5 (mGluR5) encephalitis is another type of AE which is extremely rare worldwide. Only 12 cases have been reported so far. The clinical features of mGluR5 antibodies have been reported in only 11 patients. The observed neurologic manifestations were behavior or personality/mood changes, altered cognition, sleep disturbances, seizures, decreased level of consciousness, movement disorders.^[4]^ However, the effects of mGluR5 antibodies are not clear.

The coexistence of anti-LGI1 and anti-mGluR5 encephalitis has been rarely reported. Herein, we report, to our knowledge, the first case of a 65-year-old Chinese female presenting with two rare coexisting autoimmune syndromes: anti-LGI1 and anti-mGluR5 encephalitis.

## 2. Case report

**Figure 1.**
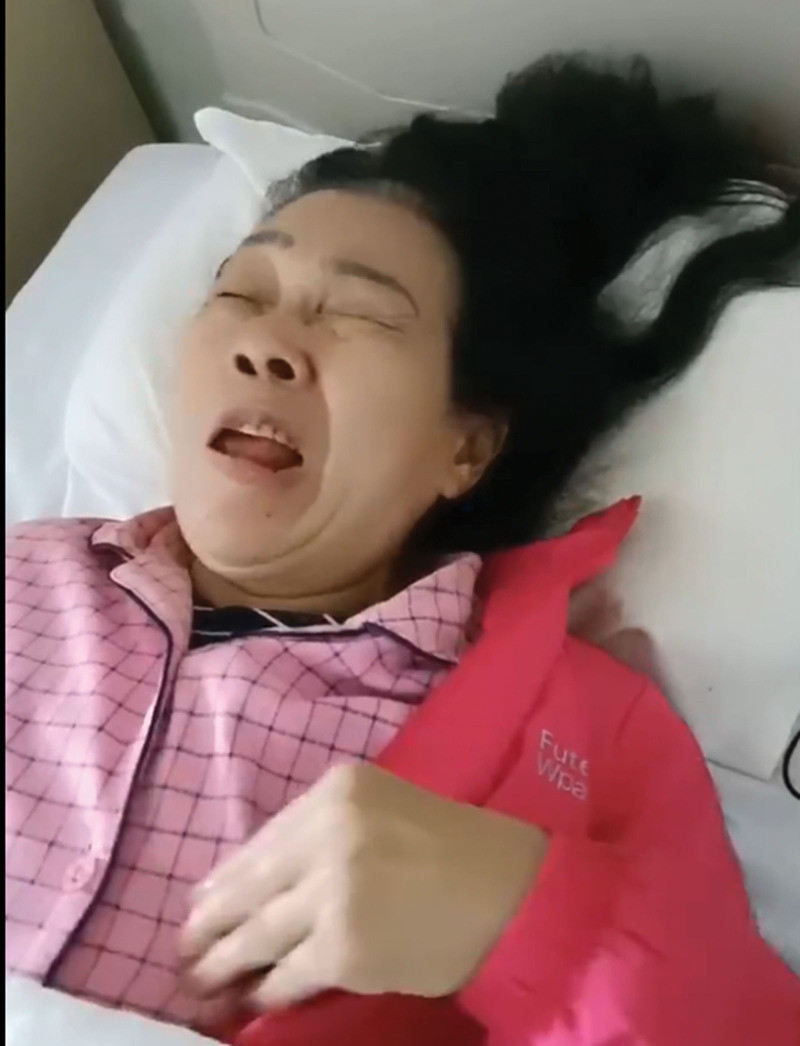
Left faciobrachial dystonic seizure.

**Figure 2.**
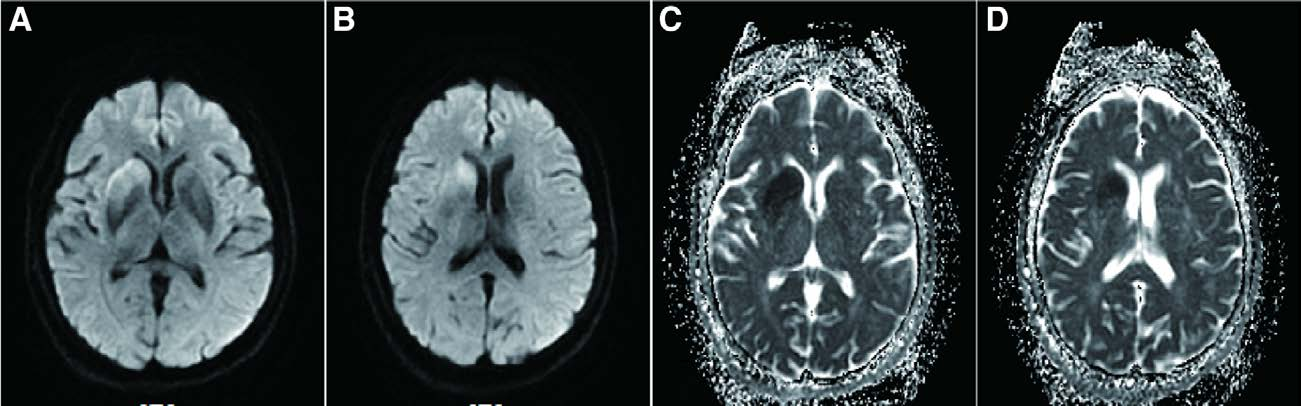
Diffusion weighted imaging (DWI) revealed diffusion restriction at the right putamen and caudate nucleus (A and B) and apparent diffusion coefficient (ADC) at the corresponding position (C and D).

A 65-year-old woman without underlying disease was admitted to the emergency department with the symptoms of sudden onset left faciobrachial dystonic seizures and unresponsive (Fig. 1). She was without fever, lateral limb weakness, dizziness, mental disorder and loss of consciousness. Cranial computed tomography (CT) was normal. Diffusion weighted imaging revealed diffusion restriction at the right putamen and caudate nucleus and apparent diffusion coefficient at the corresponding position (Fig. 2). Upon admission on April 13, neurological examination revealed left faciobrachial dystonic seizures, unresponsive and a positive left Babinski sign. The symptoms was continuous for 5 hours and she was diagnosed with ischemic stroke, so we administered 1 million units of urokinase for intravenous thrombolysis. Thrombolytic therapy went smoothly. There was no unresponsive and involuntary movement of left upper extremity, the frequency of convulsion on her left face was obviously decreased after thrombolytic therapy. However, 5 hours after thrombolysis, the patient had sudden onset twitching of left lower extremity and then spread to right lower extremity. The symptom appeared intermittently and accompanied confusion in severe cases.

**Figure 3.**
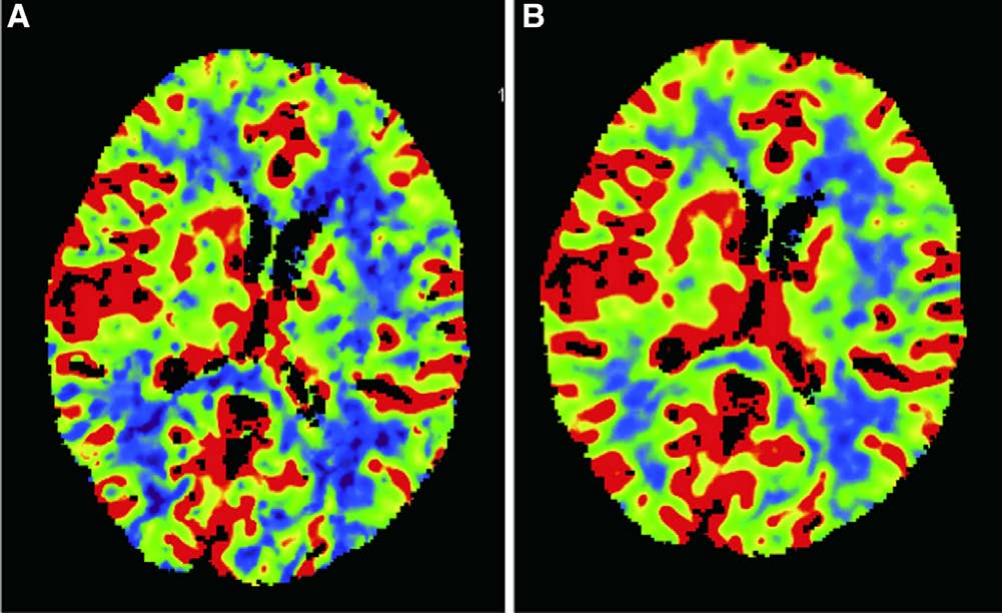
Computed tomography perfusion (CTP) showed hyperperfusion in the right basal ganglia.

**Figure 4.**
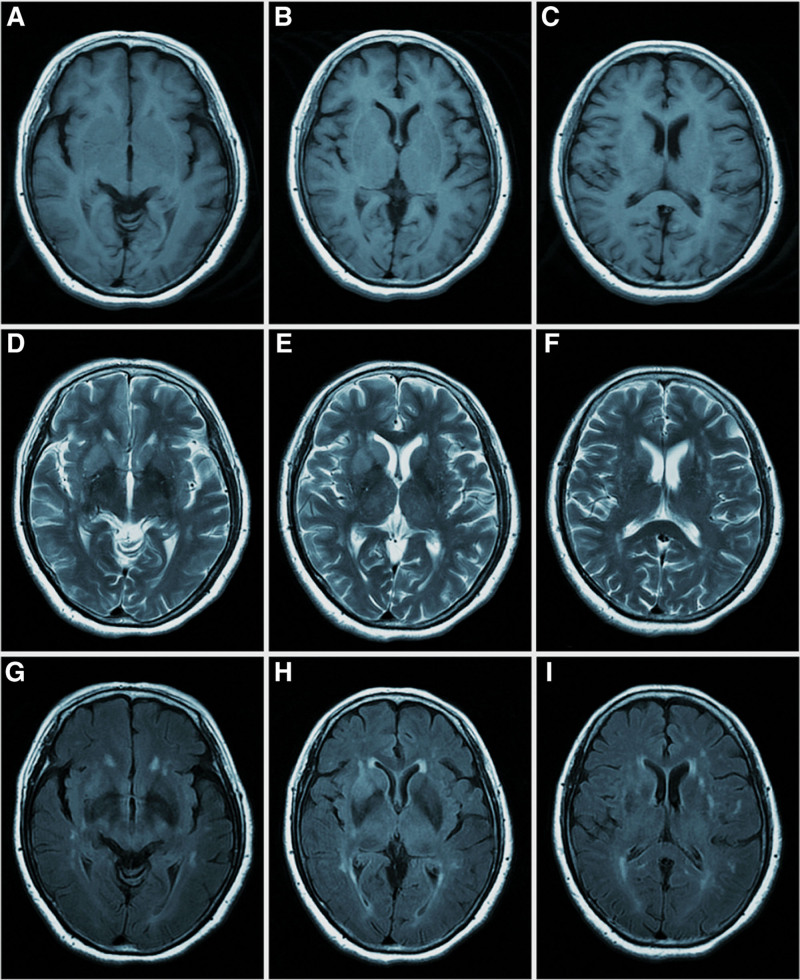
MR scan of the brain revealed abnormal signals in the right side of the caudate nucleus and putamen, with T1-weighted sequence of hypointensity (A–C), T2-weighted sequence of hyperintensity (E and F), FLAIR sequence of hyperintensity (G–I).

**Figure 5.**
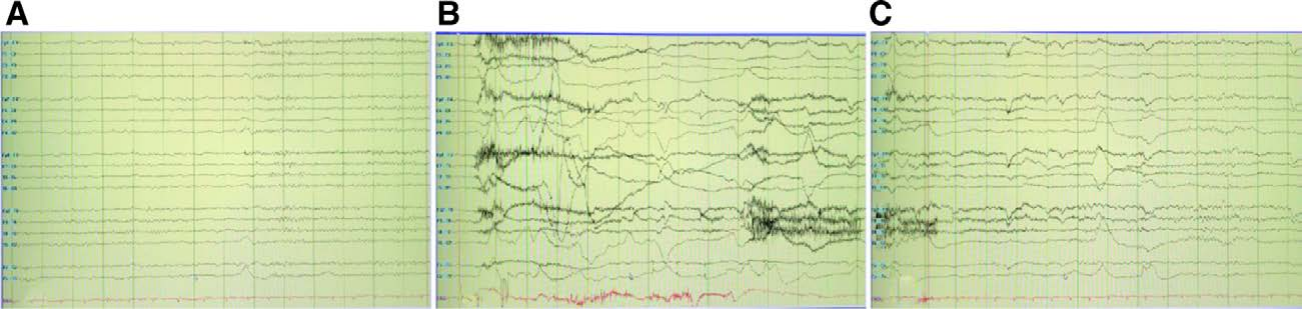
(A) Video electroencephalography (EEG) shows normal rhythm before the attack of FBDS. (B) Following myogenic artifact associated with a faciobrachial dystonic seizure. (C) After that diffuse flattering of the background activity. FBDS = facial-brachial dystonia episodes.

Therefore, we further conducted cranial computed tomography perfusion imaging, which revealed hyperperfusion in the right basal ganglia (Fig. 3). No significant abnormalities were showed on computed tomography angiography. MR scan of the brain revealed abnormal signals in the right side of the caudate nucleus and putamen, with T1-weighted sequence of hypointensity, T2-weighted sequence of hyperintensity, FLAIR sequence of hyperintensity (Fig. 4). The electroencephalography was abnormal (Fig. 5). Electromyography showed multiple peripheral nerves damage. Accordingly, we gave the patient levetiracetam (500 mg bid) and sodium valproate (0.5 g bid) to anti-epileptic.

**Figure 6.**
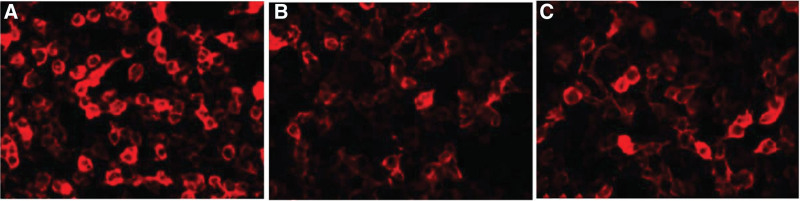
(A) The expression of LGI1 antibody (1:100) in serum. (B) The expression of mGluR5 antibody (1:10) in serum. (C) The expression of LGI1 antibody (1:30) in cerebral spinal fluid (CSF). LGI1 = leucine-rich glioma-inactivated 1, mGluR5 = metabotropic glutamate receptor 5.

Blood tests showed levels of potassium (3.2 mmol/L), creatine kinase (259.1 U/L), CK-MB (20.2 U/L), myoglobin (410 ng/mL), alpha fetoprotein (7.14 ng/mL). The routine blood, thyriod function, antinuclear antibodies, antinuclear antibody spectrum, hepatitis, vasculitis screening, coagulation test, HIV, TP, immune factor, erythrocyte sedimentation rate, cardiac ultrasound, gynecological ultrasound, chest CT, abdominal CT showed no obvious abnormalities. The routine cerebral spinal fluid (CSF) results on April 18 were normal. The test of autoimmune-associated encephalitis antibody showed seropositive antibodies of LGI1 (1:100) and mGluR5 (1:10), CSF of the LGI1 (1:30) (Fig. 6). The patient’s tests strongly supported the diagnoses of autoimmune encephalitis, therefore, she was treated with glucocorticoid (1000 mg × 3 days → 500 mg × 3 days → 240 mg × 3 days → 120 mg × 3 days) intravenous drip, then 60 mg oral. The treatment of potassium and calcium supplementation and gastric mucosa protection were also given at the same time. We also gave her the therapy of immunoglobulin (25 g q.d) for 5days. She had no recurrence of left faciobrachial and lower extremity dystonia. She was treated with meprednisone (60 mg q.d), levetiracetam (500 mg bid) and sodium valproate (0.5 g bid) after discharge and was told to reduce the meprednisone in dose of 5 mg every two weeks. She did not complain of any uncomfort until July 21.

## 3. Discussion

Here we report the first case of AE with coexistent serum anti-LGI1 and anti-mGluR5. The 65-year-old female, with onset, mainly manifested facial-brachial dystonia episodes (FBDS), unresponsive, refractory hypokalemia, abnormal diffusion weighted imaging, MRI and electroencephalography, and positive anti-LGI1 and anti-mGluR5 in serum, positive anti-LGI1 in CSF. Our patient present a good response to the intravenous methylprednisolone and intravenous immunoglobulin therapy. She did not complain of any uncomfort until July 21.

LGI1 is mainly expressed in hippocampus and neocortex which is a transsynapsin secretory protein, it connects presynaptic epilepsy-related ADAM23 to postsynaptic ADAM22. Anti-LGI1 encephalitis is the second most common AE after anti-NMDAR encephalitis which is a type of VGKC-complex antibody encephalitis and present with limbic encephalitis.^[1]^ It is characterized by FBDS and refractory hyponatremia in addition to the cognitive impairment, epilepsy, and psychiatric symptoms common in limbic encephalitis. Subtle focal seizures (66%) and FBDS (47%) mostly occurred before onset of memory disturbance.^[1]^ Only about 5% to 10% of anti-LGI1 encephalitis patients had tumors, and the most common tumor is thymoma. Normal CSF routine analysis showed in 75% cases. The brain MRI may show bilateral or unilateral abnormal signal in the medial temporal lobe or basal ganglia, especially in T2-weighted sequence of hyperintensity, or no obvious abnormality,^[2]^ about 23% cases, hyperintensities extended to the amygdala.^[1]^

Refractory hyponatremia is reported in up to 65%,^[1]^ probably in association with a syndrome of inappropriate antidiuretic hormone secretion due to the presence of LG1 receptors in the hypothalamus and kidney.^[5]^ However, our patient had hypokalemia rather than hyponatremia. Anti-LGI1 AE with hypokalemia has been rarely reported, and, to the best of our knowledge, there is only 3 cases documented until date.

One finding is that anti-LGI1 AE can occur with PNHS. Recent researches have proved that autoimmune mechanisms play a vital role in the pathophysiology of PNHS, serum CASPR2 and LGI1 antibodies may be pathogenic for primary PNHS, mainly through interfering with the function of the VGKC-complex.^[3]^ After-discharges are considered electrophysiological manifestations that are effective in diagnosing PNHS.^[6]^ However, our patient’s electromyography showed peripheral nerve damage.

Glutamate receptors (GluRs) are the main mediators of excitatory synaptic transmission in the brain.^[4]^ GluRs can be classified as ionotropic glutamate receptor and metabotropic glutamate receptor. Metabotropic glutamate receptor are coupled to G proteins and activate intracellular signaling.^[4]^ Anti-mGluR5 encephalitis is another type of AE which is extremely rare worldwide. Only 12 cases have been reported so far. The observed neurologic manifestations were behavior or personality/mood changes (91%), altered cognition (91%), sleep disturbances (64%), seizures (55%), decreased level of consciousness (55%), movement disorders (45%).^[4]^ However, the effects of mGluR5 antibodies are not clear.

With the expansion of neuronal antibody repertoire and the increase of confirmed cases, the number of multiple antibody positive cases has gradually increased, which has attracted the attention of neurologists and researchers. However, the positivity of dual auto-antibody in autoimmune encephalitis is rare. The co-existence of LGI1 antibody and other antibodies is extremely rare. LGI1 is more commonly co-expressed with CASPR2 and can manifest as Movan syndrome. Up to date, here we report the first case of AE with coexistent serum anti-LGI1 and anti-mGluR5.

Early recognition of AE is crucial, specific autoantibodies are associated with corresponding syndromes. The coexistence of dual anti-neuronal antibodies can present the superposition syndrome, which often needs to be analyzed according to the specific antibody type and clinical manifestations. The antibodies may be distinguished as pathogenic markers or concomitant antibodies. Accordingly, the main manifestations of our patient are FBDS and unresponsive, more similar to clinical syndrome of anti-LGI1 encephalitis.

The coexistence of multiple neuronal antibodies suggests a larger underlying malignancy. Only about 5% to 10% of anti-LGI1 encephalitis patients had tumors, and the most common tumor is thymoma. Tumor is reported in up to 70% of anti-mGluR5 AE, more common in hodgkin lymphoma. However, the tumor screening of our case showed negative. The patient was advised to take tumor markers during follow-up.

AE is emerging as an important and relatively common cause of encephalitis. The discovery and expansion of the anti-neuronal antibody repertoire has provided a series of important diagnostic markers for autoimmune encephalitis, while many new problems and challenges have emerged. The clinical significance and mechanism of the coexistence of multiple anti-neuronal antibodies remain to be further studied.

## Author contributions

**Conceptualization:** Tiantian Huo, Xintong Luo, Jingru Zhao, Tianjun Wang.

**Data curation:** Tiantian Huo, Xintong Luo.

**Formal analysis:** Tiantian Huo.

**Investigation:** Jinghong Chen.

**Writing – original draft:** Tiantian Huo.

**Writing – review & editing:** Tiantian Huo.
